# Multi-source detection based on neighborhood entropy in social networks

**DOI:** 10.1038/s41598-022-09229-2

**Published:** 2022-03-31

**Authors:** YanXia Liu, WeiMin Li, Chao Yang, JianJia Wang

**Affiliations:** 1 School of Computer Engineering and Science, Shanghai University, Shanghai, 200444 China; 2 Shanghai Lixin University of Accounting and Finance, Shanghai, 201209 China

**Keywords:** Computational science, Computer science

## Abstract

The rapid development of social networking platforms has accelerated the spread of false information. Effective source location methods are essential to control the spread of false information. Most existing methods fail to make full use of the infection of neighborhood information in nodes, resulting in a poor source localization effect. In addition, most existing methods ignore the existence of multiple source nodes in the infected cluster and hard to identify the source nodes comprehensively. To solve these problems, we propose a new method about the multiple sources location with the neighborhood entropy. The method first defines the two kinds of entropy, i.e. infection adjacency entropy and infection intensity entropy, depending on whether neighbor nodes are infected or not. Then, the possibility of a node is evaluated by the neighborhood entropy. To locate the source nodes comprehensively, we propose a source location algorithm with the infected clusters. Other unrecognized source nodes in the infection cluster are identified by the cohesion of nodes, which can deal with the situation in the multiple source nodes in an infected cluster. We conduct experiments on various network topologies. Experimental results show that the two proposed algorithms outperform the existing methods.

## Introduction

The rapid popularity of social media enables people to obtain some information easily and quickly from social networks^1^. The development of social platforms and internet technology have brought some issues while making our lives more convenient. For example, unverified content in social networks can spread rapidly in the network^2^. This affects people’s lives and brings great losses to society. The propagation of infectious^3^ and computer viruses on the internet^4^ can also be led to millions of destructive events. It is crucial to identify the diffusion source to control the spread of this negative information. Locating and tracking the sources is helpful to control the dissemination of information from the source. It can reduce the harm of rumor by controlling the source, cutting off the critical path of rumor propagation.

In recent years, researchers have conducted a series of works on source detection in social networks and proposed a large number of source location methods. Some current methods assume that there is only one source node in the network. For example, Shah et al.^5^studied the problem of source detection earlier and proposed the rumor center method. Some studies have adopted centrality measures for source location, such as distance center^6^, betweenness center^7^, Jordan center^8,9^, degree center^10^ and so on. Recently, some researchers have studied some central metrics in the network, such as Meghanathan et al.^11^ proposed a computationally light neighborhood-based bridge node centrality tuple to identify the bridge nodes of a network. Rajeh et al.^12^ developed a community-aware centrality metric by exploiting the community structure features of the network. Other methods proposed for a single source location, such as back propagation^13^, maximum likelihood estimation^14^ etc. However, due to the complexity of the network structure and the randomness of information diffusion, there may be multiple source nodes in the network. The diffusion process of different sources usually interacts with each other, which produces uncertainty in the propagation. The single source detection algorithm cannot be applied in the multi-source diffusion network. It faces great challenges to find out the tools to identify the diffusion source.

For the multiple sources location, the main research methods focus on network partition^6,15,16^ and ranking^17–19^. In addition to the two methods, other works also propose a new way to solve the multi-source location, such as approximation-based methods^20,21^, and heuristic methods^22,23^. To handle the problem in multi-source localization, we propose two algorithms to make full use of the infected neighborhood information of nodes. The first is neighborhood entropy. This method locates the core source node through the neighborhood information of the node. According to the core source nodes, the infection network is divided into multiple infection clusters. In each infection cluster, the source nodes are located according to the cohesion of nodes. This method solves the single source node problem in a network partition. The main contributions of this paper are as follows:

- We define the neighborhood entropy of a node. The infected adjacency entropy of a node is calculated according to the infection possibility of the node to the neighborhood nodes. The infection intensity entropy relates to the impact of the uninfected neighbors. The core source nodes depend on the neighborhood entropy relationship between the nodes.
- We propose a multi-source location algorithm based on infection cluster. With the core source nodes in our hands, we secondly study a two-stage infection cluster partition algorithm. In each infection cluster, the condensation node can find with the cohesion of the node. This further improves the accuracy of multiple source location.
- We compare the proposed methods with the state-of-the-art models on several synthetic and real networks. Experimental results show the effectiveness of the proposed methods.

## Related work

The main research methods of multi-source detection include network partition, ranking, and approximation. Here, we mainly introduce the research status of multi-source localization methods in social networks.

The partition-based methods transform the problem about multi-source location into a single source location. Generally, the network is partitioned in some way that the single source location method can be used to identify in each partition. Zang et al.^15^ proposed a community division method to identify multiple sources in each community. They also studied the multi-source localization problem by approximating the multiple independent single-source localization^16^. This adopted the divide-and-conquer strategy to solve the multi-source detection problem in the SIR model. Jiang et al.^6^ proposed the K-center method to identify a single source node. This converts the original diffusion probability network into a distance network. It is difficult to apply in the real world with the assumption that the infection probability is known. Zhu et al.^17^ proposed the optimal Jordan coverage algorithm. Syed shafat et al.^18^ proposed a source detection algorithm with the age exemption and prominence (EPA). They calculated the age of nodes by considering the prominence of nodes in their neighbors. Wang et al.^19^ proposed the method of overlapping community detection with the topological potential and infection neighbor bias for source localization. The partition-based methods need to select the initial partition center, it will affect the final source location effect.

The ranking-based methods estimate the value of each node and then select the first *k* nodes with a higher value as the source node. This kind of method requires that the *k* value is given in advance, which is difficult to obtain in real situations. Nguyen et al.^20^ proposed an algorithm in reverse diffusion. They apply ranking and optimization to find the largest *k* suspicious nodes in the network. Fiorti et al.^21^ proposed a dynamic age approach. The spectrum technology is used to identify the source by calculating the reduction of the maximum eigenvalue of the adjacency matrix after removing the node. This method can identify the source node well when the graph is similar to the tree, but it is not suitable for large-scale networks. The approximation method can find the approximate solution for source localization by minimizing and maximizing the proposed objective function. Prakash et al.^22^ constructed the NTSLEUTH model to search multiple source nodes, which uses the principle of minimum description length to generate a set of source nodes. Zhang et al.^23^ considered detecting multiple rumor sources from the perspective of certainty. They modeled as an analytic set (SRS) problem and proposed a polynomial-time greedy algorithm for finding the minimum SRS in general networks.

The source location problem is similar to the influence maximization problem^24–27^ and the super spreader problem^28^ of finding nodes, but these problems have certain differences. Firstly, they have different goals. The influence maximization problem and super spreader problem are to select *K* nodes in the network under a given budget condition *K*, to maximize the influence expansion of these *K* nodes. The source location problem is to identify single or multiple sources of information dissemination according to the network topology and the infection of nodes in a given dissemination network. However, the source location problem needs to accurately locate source nodes, and the number of source nodes also needs to be determined. Secondly, the evaluation criteria are different. In the influence maximization problem and super spreader problem, the number of affected nodes is the most important standard to measure this kind of problem, while in the source location problem, the recall and precision of source nodes are the standards to measure the source location algorithm.

## Methods

### Information propagation model

Given an undirected network $G=\left( V, E\right)$, *V* is the set of nodes, *E* is the set of edges, and each edge is $\left( u, v\right)$, where $u, v\in V$. We assume that information diffusion follows the diffusion dynamics in the classical SI model^29^. The SI model is widely used because of its simple model and can well simulate the dynamic characteristics of information diffusion. However, under the condition of complete observation, other models can still be applied, such as SIR model. This paper only gives the diffusion dynamics and experimental display of SI model. In this model, each node $u\in V$ has two possible states at discrete time *t*: susceptible state (*S*) and infected state (*I*). At the time slot, each infected node will try to independently infect its neighbor node with probability $P_{uv}$, where $P_{uv}$ represents the infection probability from node *u* to node *v*. If a node is infected, it will remain in the state all the time. Then it spreads the information to its susceptible neighbor nodes and continues to propagate in the new network topology at the next time. The probability $\lambda (v, t)$ is defined as the node *v* infected by the infected neighbor node at time *t*, $\lambda \left( v, t\right) =1-\prod _{u\in N_{v}(t-1)}\left[ 1-P_{uv}\cdot P_{I}(u,t-1)\right]$, where $N_{v}\left( t-1\right)$ represents the set of neighbor nodes of node *v* at time $t-1$. The probability that node *v* is in the infected state at time *t* can be expressed as $P_{I}\left( v, t\right) =\lambda (v,t)\cdot P_{S}\left( v,t-1\right) +P_{I}\left( v,t-1\right)$.

#### Problem definition

This paper assumes that information generates from *m* nodes. We represent the source node set as $S=\left\{ s_{1},s_{2},\ldots ,s_{m}\right\}$, where *m* is a constant and satisfies $m\ll N$, $S\subset G$. The source node set *S* starts the diffusion based on the SI model at an unknown time slot *t*. After a certain period, we can observe $N_{I}$ infected nodes, where $\left| N_{I}\right| \gg \left| S\right|$. These infected nodes form the infected subgraph snapshot $O\subset G$. The task of this paper is to locate the source node set $S\subset V$ of the initial diffusion with the infected subgraph *O* and the original network structure *G*. Table 1 provides a brief description of the notations used in this paper.

**Table 1:** Notations.

Notation	Description
*N*(*i*)	Neighbor node set of node *i*
$P_{uv}$	Infection probability from node *u* to node *v*
$\lambda (v,t)$	Probability of node *v* being infected by neighbor nodes at time *t*
$V_{I}$	All infected nodes
$I_{i}$, $U_{i}$	Infected (Uninfected) neighbor nodes of node *i*
$\xi _{i}$	Infection intensity of node *i*
$\eta _{i}$	Infection degree of node *i*
$\psi _{i}(j)$	Contribution of node *j* to node *i*
$IE_{i}$	Infection intensity entropy of node *i*
$AE_{i}$	Infection adjacency entropy of node *i*
$NE_{i}$	Neighborhood entropy of node *i*
$C_{s}$	Core convex set
$Sim(n_{1},n_{2})$	The similarity between node $n_{1}$and node $n_{2}$
$\delta _{i}$	Cohesion strength of node *i*
$\hat{S}$	Predicted source nodes

### Multi-source location with neighborhood entropy

In this section, we propose a multi-source location algorithm with neighborhood entropy. The possibility of a node is the source node measured by the neighborhood entropy. The greater the neighborhood entropy, the more infection information the node carries in the diffusion network. If a node carries the information larger than the neighbors, it is more likely to be the source. In an infection subgraph, a node will be affected by two factors. One is the infected neighbor nodes, the other is those uninfected neighbor nodes which has a certain weakening effect.

#### Infection intensity entropy

In a network, the information spreads from infected nodes to uninfected neighbors. The earlier a node is infected, the longer it takes to infect its neighbor nodes. This generates more of the number of infected neighbors and provides a higher probability to be a propagation source. In other words, if two nodes have the same number of infected neighbors, the node with more uninfected neighbors is less likely to be the source node. We use the infection intensity to define the influence of uninfected nodes, as shown in Definition 1.

##### Definition 1

(*Infection intensity*) The infection intensity of a node is used to measure the effect of uninfected neighbors on the node. The smaller the proportion of uninfected nodes, the higher value of the infection intensity, as shown below

1 $$\begin{aligned} \xi _{i}=\frac{\left| N\left( i\right) \right| -\left| U_{i}\right| }{\left| N\left( i\right) \right| }\times \frac{1}{1+e^{-|N(i)|}}, \end{aligned}$$

where $|U_{i}|$ represents the number of uninfected neighbor nodes of node *i*, *N*(*i*) represents the number of all neighbor nodes of node *i*. The second part $\frac{1}{1+e^{-|N(i)|}}$ is to eliminate the influence of the node degree.

##### Definition 2

(*Infection intensity entropy*) The infection intensity entropy is defined by the logarithm of the infection intensity. It is used to measure the impact of the node’s uninfected neighbors on the node, as shown below

2 $$\begin{aligned} IE_{i}=-\xi _{i}log_{2}\xi _{i}. \end{aligned}$$

#### Infection adjacency entropy

In an infected network, we can measure the infection information carried by the neighbor nodes. The longer the diffusion time, the more nodes will be infected in the neighborhood. For a node, the probability of the node infecting its neighbors can be measured by its neighborhood, which is the infection degree as shown in Definition 3.

##### Definition 3

**Figure 1:**
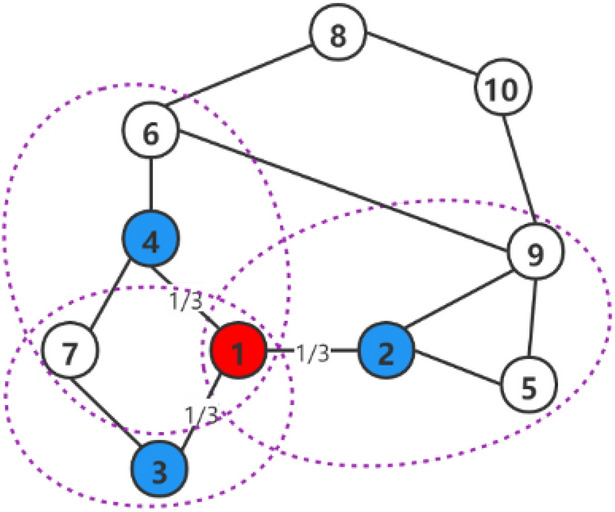
Diffusion network. Red node 1 represents the source node, blue node is the domain node of node 1, and each dotted box represents the domain of the blue node.

(*Infection degree*) The infection degree of a node is determined by its neighbor nodes, indicating the possibility of the node is the parent of all neighbors. Here, the infection degree of node *j* is given as

3 $$\begin{aligned} \eta _{j}=\sum _{t\in N(j)}\frac{1}{|N(t)|}\cdot \xi _{t}, \end{aligned}$$

where *N*(*j*) represents the neighbor set of node *j*. For example, in Fig. 1, the neighbor node of node 2 is node {1, 5, 9}, so the infection degree of node 2 is $\eta _{2}=\frac{1}{|N(1)|}\cdot \xi _{1}+\frac{1}{|N(5)|}\cdot \xi _{5}+\frac{1}{|N(9)|}\cdot \xi _{9}=\frac{1}{3}\cdot \xi _{1}+\frac{1}{2}\cdot \xi _{5}+\frac{1}{4}\cdot \xi _{9}$.

In the process of information diffusion, the source nodes will infect their neighbors with a probability. Each infected neighbor will make certain contributions to the target node. We define the contribution degree, as shown in Definition 4.

##### Definition 4

(*Contribution degree*) The contribution degree is used to measure the probability that node *i* is regarded as the parent of its neighbors. The more contribution makes to its neighbor node, the higher likelihood the node to be the source. The contribution of node *i* to node *j *can be expressed as

4 $$\begin{aligned} \psi _{i}(j)=\frac{\frac{1}{|N(i)|}}{\eta _{j}}. \end{aligned}$$

##### Definition 5

(*Infection adjacency entropy*) The infection adjacency entropy of a node is determined by the contribution to its neighbor nodes, as shown below

5 $$\begin{aligned} AE_{i}=-\sum _{j\in N(i)}\psi _{i}(j)log_{2}\psi _{i}(j). \end{aligned}$$

##### Definition 6

(*Node neighborhood entropy*) The neighborhood entropy of a node is composed of infection neighborhood entropy and infection intensity entropy. To reduce the information carried by uninfected neighbor nodes, the infection intensity entropy is subtracted from the infection adjacency entropy to obtain the node neighborhood entropy, which can be expressed as

6 $$\begin{aligned} NE_{i}=AE_{i}-\alpha \times IE_{i} , \end{aligned}$$

where $\alpha$ represents the weight factor of infection intensity entropy. Through experiments, we find that when $\alpha =4$, the source location effect is the best, so this paper sets $\alpha$ to 4.

The greater the neighborhood entropy of a node, the more infection information will carry. Based on this idea, if the neighborhood entropy of a node is greater than that of all neighbor nodes, it is a core convex node. All core convex nodes form the core convex set as expressed as

7 $$\begin{aligned} C_{s}=\bigcup _{i\in N_{I}}\left\{ NE_{i}>Max_{j\in N(i)}NE_{j}\right\} . \end{aligned}$$

Following the above concepts, the specific process of source localization with neighborhood entropy (SLBNE) can be achieved in Algorithm 1. The time complexity of SLBNE relates to the number of nodes and the number of neighbors, which is *O*(*ND*), where *D* is the degree of the largest degree node and *N* is the number of all infected nodes in the network.
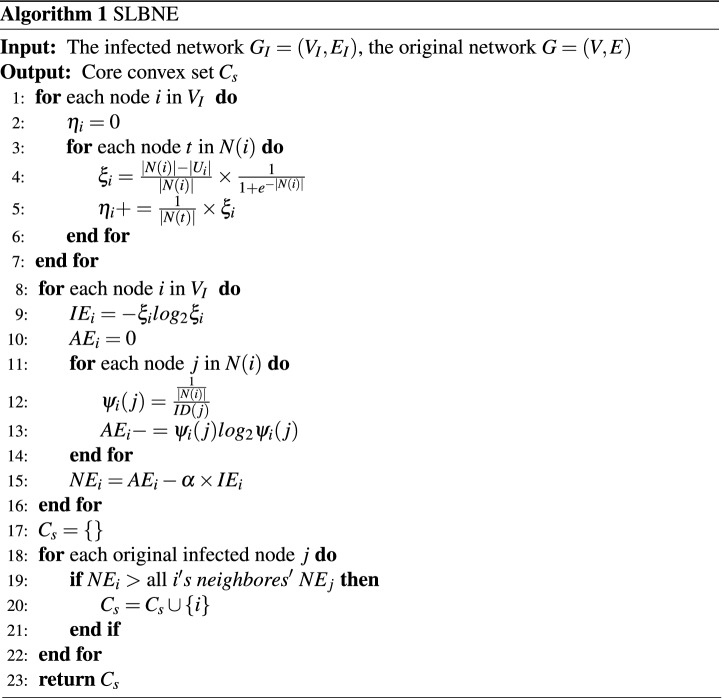

### Multi-source location with infection cluster

SLBNE can locate the source node with low computational complexity. However, with the increase in the number of source nodes, SLBNE may cause the incomplete location of source nodes. SLBNE locates the source node by comparing the neighborhood entropy of the node and its neighbor nodes according to entropy convex. If two source nodes are neighbors, one of the two nodes cannot be located using SLBNE. Therefore, based on SLBNE, by dividing the network, we use node cohesion to locate other undetected source nodes. Compared with SLBNE, SLBIC increases the computational complexity, but the corresponding source localization recall has been improved, which is suitable for the scene with location recall as the main target when there are a large number of source nodes.

#### Infection cluster division

A core convex set containing *r* convex nodes is obtained by neighborhood entropy. Here we take the node as the center in the core convex set and divide all non-core nodes into *r* infection clusters. The partition includes two steps. The first step is to divide the direct neighbors of the core node. We calculate the similarity between direct neighbors and each core node in Eq. (8). The node will be divided into a cluster with the most similar core node.

8 $$\begin{aligned} Sim(n_{1},n_{2})=\frac{Com(n_{1},n_{2})}{|N(n_{1}) \bigcup N(n_{2})|}+\sum _{i\in N(n_{1})}\sum _{j\in N(n_{2})}\frac{Com(i,j)}{|N(i)\bigcup N(j)|}, \end{aligned}$$

where $Com(n_{1},n_{2})$ represents the number of common neighbors of nodes $n_{1}$ and $n_{2}$. $N(n_{1})$ represents the number of neighbor nodes of node $n_{1}$.

The second step is to divide all nodes that have not yet entered the infection cluster. According to the edge connection between the node and all infection clusters, the node will be divided into infection clusters with more edges. If a node has the same number of connecting edges with multiple infection clusters, the node belongs to infection clusters by considering the overlapping situation of infection clusters.

Algorithm 2 describes the specific process of infection cluster division (ICD). The time complexity for similarity calculation is *O*(*DD*), and for cluster division is *O*(*rN*). The total time complexity for ICD is $O (DD + rN)$, where *D* is the degree of the maximum degree node. *N* denotes the number of all infected nodes, and *r* represents the size of the core convex set. 
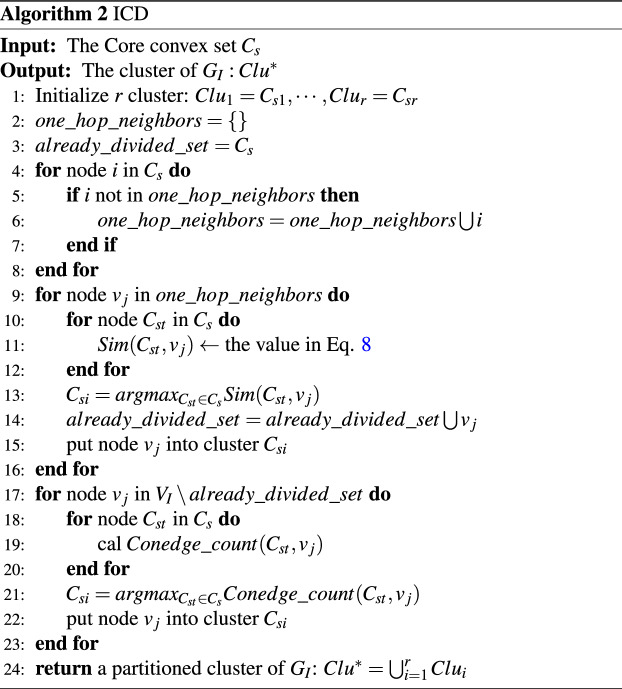


#### Source location

In real situations, there are multiple source nodes in an infection cluster. We propose a multi-source localization algorithm with the infection cluster. We calculate the cohesion of nodes in each infection cluster and select the node with the largest cohesion.

The cohesion of a node measures the centrality in the infected cluster. The sum of the path distance between a node and others in the infected cluster with the neighborhood entropy is the cohesion of the node. Firstly, we compute the shortest path distance between node *i* and all other nodes in the infected cluster. Then it is divided into different sets according to the length of paths. $path\_dic_{i}=\left\{ dis_{ij}:count_{j}\right\}$, where $dis_{ij}$ represents the distance length, $count_{j}$ is the number of paths with a distance equal to $dis_{ij}$ in the infected cluster where node *i* is resides. Then the cohesion of node *i* can be given by

9 $$\begin{aligned} \delta _{i}=\sum _{j\in path\_dic. keys}\frac{M_{dis}+1-dis_{ij}}{M_{dis}+1}\times \frac{count_{j}}{APN}\times 0.5 + NE_{i}\times 0.5 , \end{aligned}$$

where $M_{dis}$ represents the longest distance between node *i* and all nodes in the infected cluster, *APN* represents the number of all possible path lengths. We define the node with the largest cohesion in the infected cluster as the condensed node, which is $CN_{i}=Max_{v_{j}\in Clu_{i} }\delta _{v_{j} }$. We can get a set of condensed nodes, expressed as $C_{s1}$.

Finally, The predicted source nodes is the union of condensed nodes and core source nodes, which can be expressed as

10 $$\begin{aligned} \hat{S} = C_{s}\bigcup C_{s1} . \end{aligned}$$

According to the above description, the specific process of source localization based on infection cluster (SLBIC) is described in Algorithm 3. The time complexity of SLBIC is $O (ND + rN)$. 
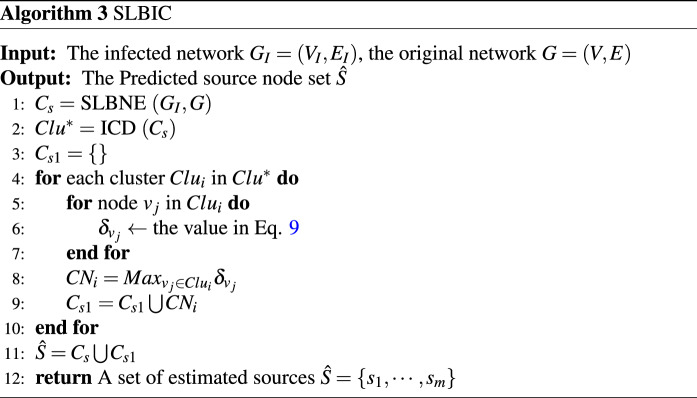


## Experimental evaluation

To evaluate the performance of two proposed algorithms, we compare to the NETSLEUTH^22^, K-CENTER^6^, TP^19^ and PCL^30^ on six data sets.

### Experiment settings

#### Datasets

We firstly introduce real networks, that is, Karate^31^, Dolphin^32^, Celegans^33^, Facebook^34^, Git^35^ and Gowalla^36^, and several synthetic networks, that is, ER and BA, as the experimental data. What is more, all real datasets are available online (http://networkrepository.com/networks.php, http://snap.stanford.edu/data/index.html). The topological properties of these networks are shown in Table 2.

**Table 2:** The topology properties of networks.

DataSet	|*V*|	|*E*|	$<d>$	$<k>$	$\varsigma$
Karate	34	78	2.41	4.60	0.570
Dolphin	62	159	3.36	5.13	0.259
Celegans	453	2025	2.66	8.94	0.646
Facebook	4039	88,234	3.69	43.69	0.606
Git	37,700	289,003	3.25	15.33	0.168
Gowalla	196,591	950,327	4.627	9.668	0.237

|*V*| and |*E*| denote the number of nodes and edges in the network, respectively. $$<d>$$ denotes the average length of all shortest paths. $$<k>$$ denotes the network average. $$\varsigma$$ denotes the average clustering coefficient of the network.

#### Parameter settings

To make a more comprehensive comparison, we select a different number of source nodes to compare. For small infection graph sizes, such as Karate, Dolphin and Celegans, the source numbers *k* are 2, 3, 5, respectively. For the larger data, such as Facebook, Git and Gowalla, the source numbers *k* are 3, 5, 8 in each case. All the experimental results run 100 independent times to ensure credibility. All infections are independent of each other. The diffusion will stop when over 30% (as the same in^37^) nodes are infected. We assume that $P_{ij}$ obeys uniformly distributed over (0, 1).

#### Efficiency measures

We use two measures to evaluate the performance of the proposed methods, namely F-score and average error distance. Consistent with previous work^37^, we choose F-score as one of the evaluation metrics. We treat the precision and recall equally and set $\beta$ to 1. Average error distance is a frequently used criterion for evaluating source location^6,19,38^.The average error distance is the average of all error distance over 100 independent runs. The distance between the estimated source and the real source is called the error distance, which can be expressed as $\Delta =\frac{1}{N_{S^{ *}}}(min_{j\in \hat{S}}dis(i,j)+\rho |N_{S^{*}}-N_{\hat{S}}|)$ where $S^{*}=\left\{ s_{1},s_{2},\ldots ,s_{m}\right\}$ represents the real source nodes, $\hat{S}$ is the estimated source nodes, $N_{S^{*}}$ and $N_{\hat{S}}$ represent the actual and the estimated number of source nodes respectively, *dis*(*i*, *j*) represents the shortest path length between node *i* and *j*, and the parameter $\rho$ is set to 0.5.

### Experimental result

In this section, we present the performance of source localization for two algorithms. For small infection graph sizes, we use SLBNE and SLBIC. For the large infection graph, considering the computational cost, we only test the performance of SLBNE.

#### Source location accuracy

**Figure 2:**
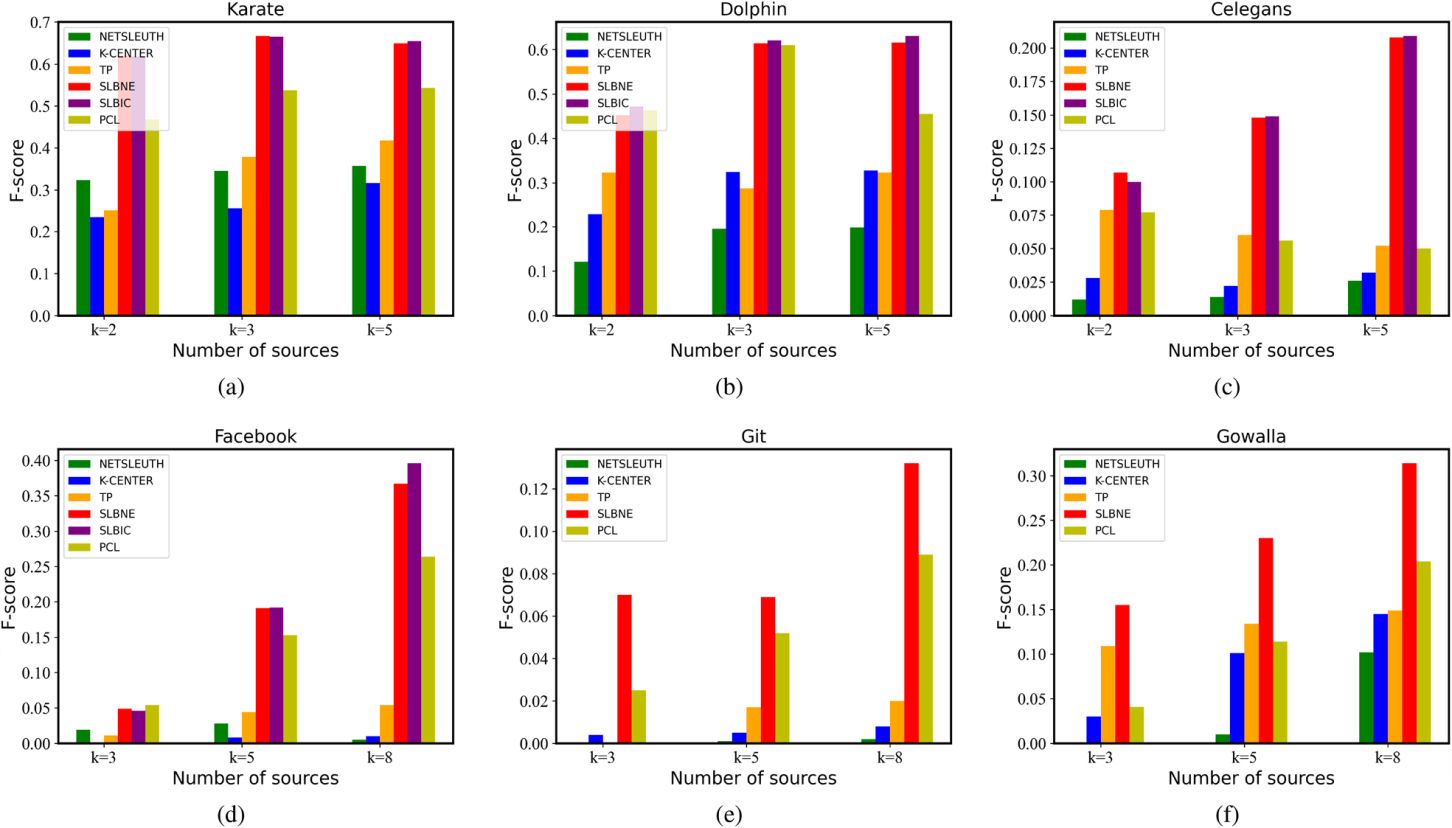
Source location accuracy.

Figure 2 shows the source location accuracy of our algorithms in the six topological networks. The experimental results show that SLBNE and SLBIC are better than all baselines. When the source node is 2, the source location performance of SLBIC is lower than that of SLBNE. With the increase of the number of source nodes, the location performance of SLBIC is the best, which indicates that when the number of source nodes is large, SLBIC will improve the source location effect.

#### Average error distance

**Figure 3:**
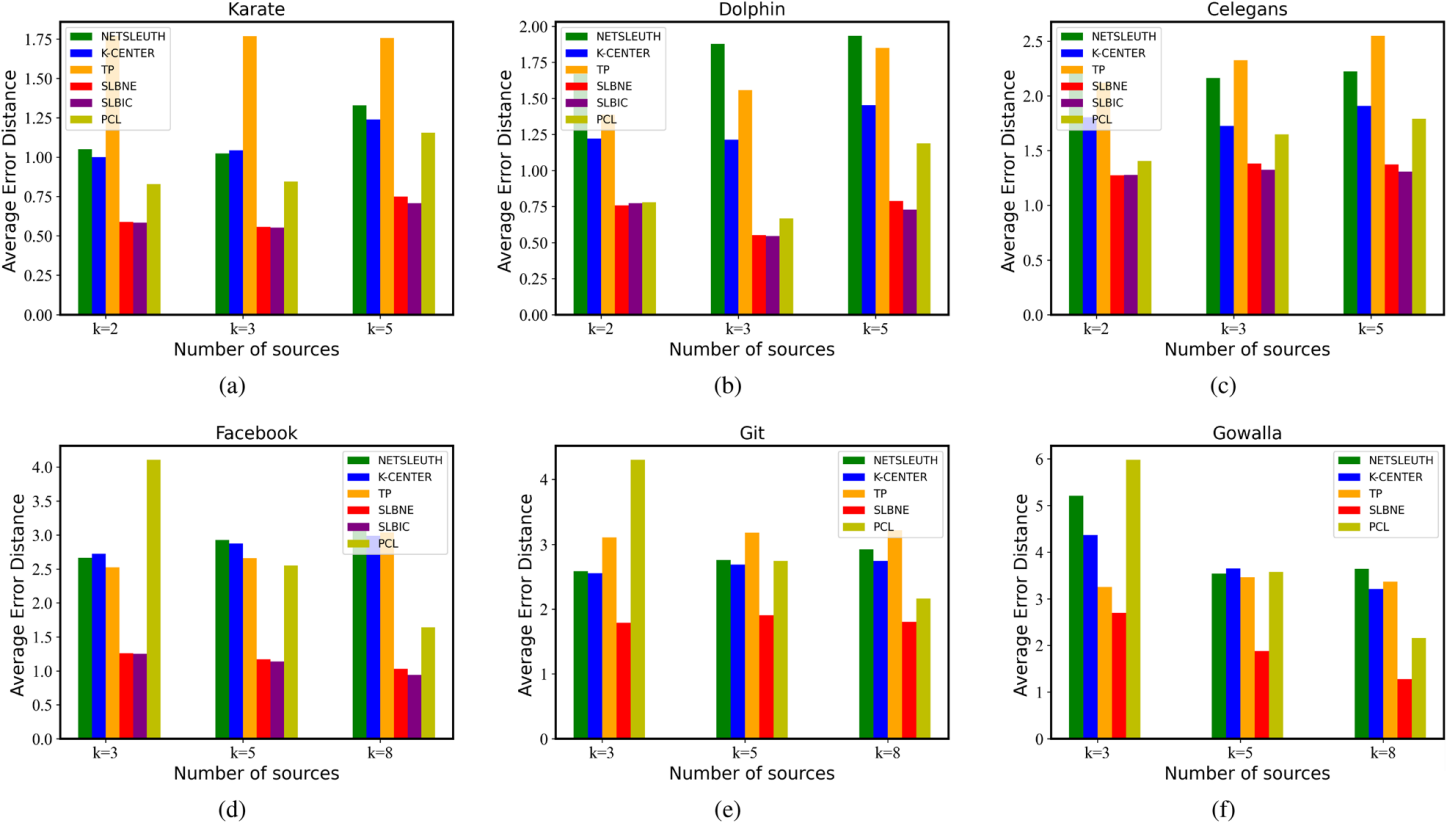
Average error distance.

Figure 3 shows the average error distance of the two proposed methods in the datasets. Since average error distance measures the average shortest path between estimated sources and true sources, the small average error distance indicates better performance. We can find that in Karate (Fig. 3a), Dolphin (Fig. 3b), Celegans (Fig. 3c), and Facebook (Fig. 3d), regardless of the number of source nodes, the average error distance of SLBIC is the minimum, followed by SLBNE. In Git (Fig. 3e) and Gowalla (Fig. 3f), the average error distance of SLBNE is significantly lower than that of other methods.

#### Number of source nodes

**Figure 4:**
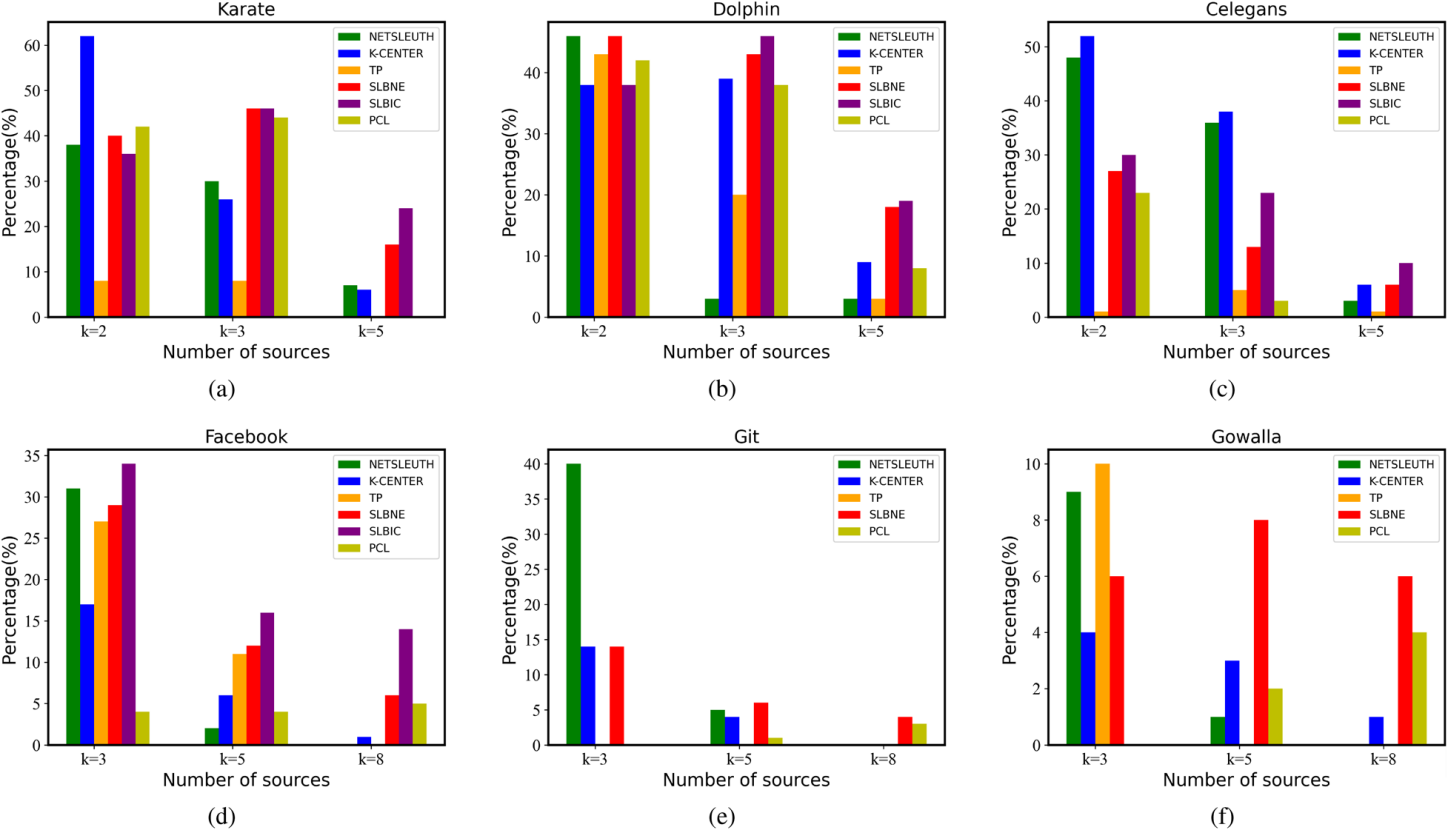
Location accuracy of the number of source nodes.

Figure 4 evaluates the accuracy of the two proposed algorithms in predicting the number of source nodes when the number of source nodes is different. We find that the two proposed algorithms can correctly predict the number of source nodes with high accuracy in most cases. When the number of source nodes is small, SLBNE predicts more accurately, while SLBIC performs better as the number of source nodes increases. NETSLEUTH and K-CENTER have higher accuracy in locating the number of source nodes when the number of source nodes is small, but from Fig. 2, we find that the source location accuracy is very low.

#### Tests in synthetic networks

**Figure 5:**
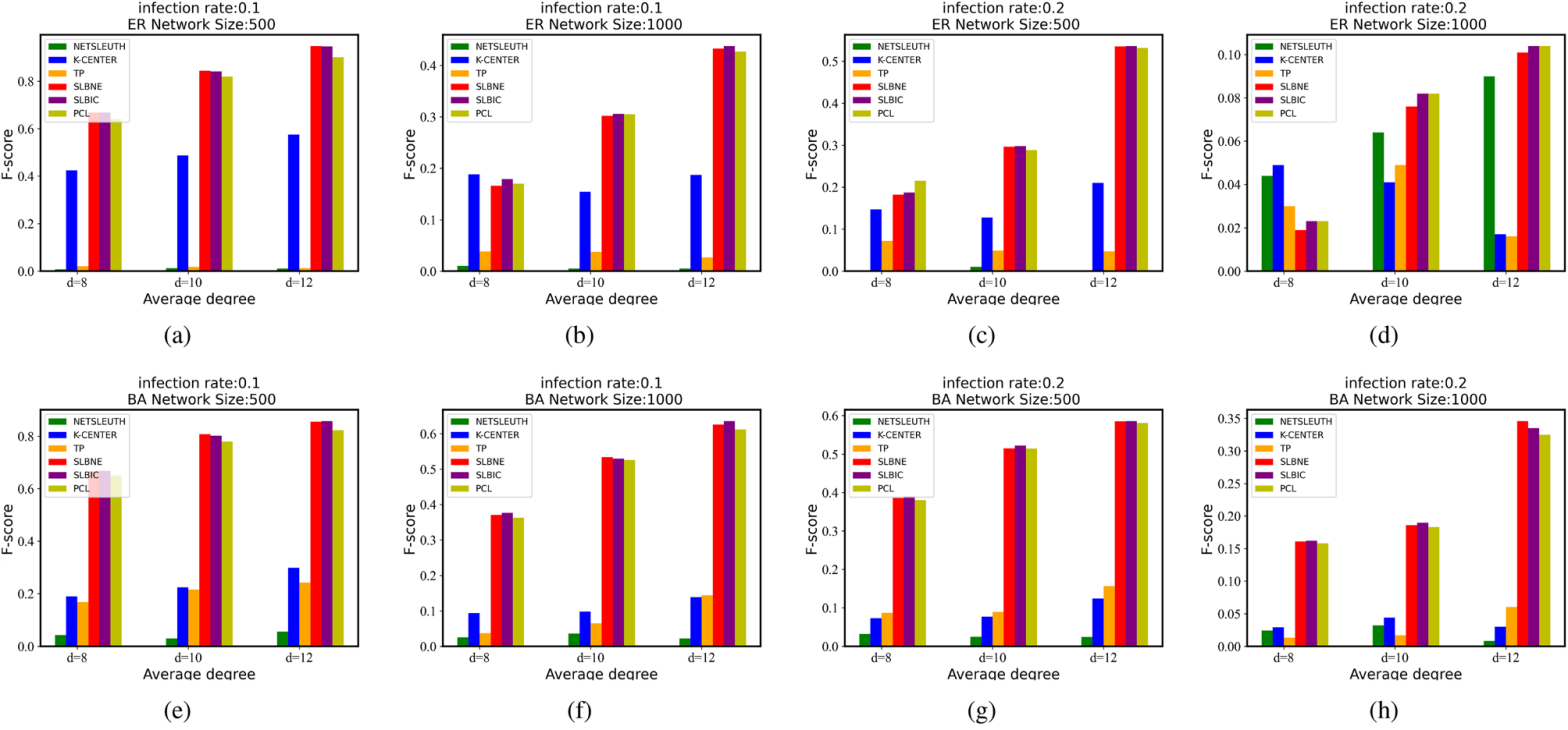
Sources localization accuracy in synthetic networks. (**a**–**d**) Results in ER network. The scale of the network is N = 500, 1000 respectively, the node infection rate is 0.1, 0.2 respectively, the average degree is 8, 10, 12 respectively. (**e**–**h**) Results in BA network. The number of source nodes is 5.

This paper has carried out some experiments in synthetic networks, namely the random (ER) network^39^, and the scale-free (BA) network^40^. From Fig. 5, we can find that SLBNE and SLBIC have the highest sources localization accuracy in all synthetic networks. In terms of infection ratio, the source location accuracy is higher when the proportion of infected nodes is low, which indicates that we can obtain better results by source location in the early stage. The source location accuracy of all algorithms changes with the increase of network scale. The smaller the network scale, the higher the location accuracy. We find that the average degree has some influence on the source location accuracy with all algorithms. When the average degree is large, the source location accuracy is higher. The dense network structure is conducive to the source location. All in all, no matter how the network changes, SLBNE and SLBIC algorithms can achieve better source location results.

## Discussion

To locate multiple source nodes when the number of source nodes is unknown, we propose a multi-source location algorithm SLBNE based on neighborhood entropy by considering the neighborhood information of nodes. Compared with baseline algorithms, the location accuracy has been improved. SLBNE makes full use of the neighborhood information of nodes, which not only considers infected neighbor nodes but also considers the influence of uninfected neighbor nodes. Because SLBNE only uses neighborhood information, the relative computational complexity is very low, and it is suitable for scenarios requiring low complexity and taking precision rate as the main target. To locate source nodes more fully, we consider the tendency of source node diffusion, and multiple source nodes spread will form multiple infection clusters. Therefore, based on SLBNE algorithm, this paper proposes a multi-source location algorithm based on infection clusters SLBIC, which locates other unlocated source nodes in each infection cluster by dividing the network. Compared with SLBNE, SLBIC improves recall. When the number of source nodes is large, SLBIC works better and is suitable for the scenario that the recall is the main target.

We verify SLBNE and SLBIC in synthetic networks (random networks and scale-free networks) and six real networks and compare the performance of these algorithms using three methods: F-score, average error distance, and location accuracy of the number of source nodes, and experiment with four benchmark methods to verify the effectiveness of the method proposed in this paper. Firstly, in the synthetic network, we analyze the source location accuracy by adjusting different parameters. Figure 5 shows that when there are few infected nodes, the source location accuracy is higher, which indicates that SLBNE and SLBIC have better effects in the early stage of information diffusion. When the average degree of the network is large, the source location accuracy is higher. Because the two proposed methods rely on neighborhood information, the denser network structure will improve the source localization effect. Experiments on six real networks show that SLBNE and SLBIC can locate the source node with higher location accuracy and lower average error distance. Compared with SLBIC, SLBNE has a better source location effect when the number of source nodes is small. With the increase of the number of source nodes, the location accuracy of SLBIC is higher than that of SLBNE, because SLBIC divides the network again based on SLBNE to locate other undetected sources nodes, which increases the recall rate. Both K-CENTER and TP transform the multi-source problem into a single source problem. However, in reality, there may be multiple source nodes in the partition, which may lead to an incomplete location of source nodes. SLBIC takes the source node located by SLBNE as the core to divide the infection cluster, which solves the problem that there may be multiple source nodes in an infection cluster. From Figs. 1 and 2, we find that the location accuracy of SLBIC is significantly higher than these two algorithms, while NETSLEUTH is not suitable for large-scale networks. The location effect of PCL is not ideal in real data sets, especially large-scale networks. In the future, we will study the influence of network structure on neighborhood entropy. We can also develop a better method to divide infection clusters, improving the performance of source location.
